# Risk of total/subtotal meniscectomy for respective medial and lateral meniscus injury: correlation with tear type, duration of complaint, age, gender and ACL rupture in 6034 Asian patients

**DOI:** 10.1186/s12893-017-0324-9

**Published:** 2017-12-05

**Authors:** Dong Jiang, Xiao Luo, Yingfang Ao, Xi Gong, Yong-jian Wang, Hai-jun Wang, Yu Miao, Nan Li, Ji-ying Zhang, Jia-kuo Yu

**Affiliations:** 1 Institute of Sports Medicine, Peking University Third Hospital, Beijing Key Laboratory of Sports Injuries, Beijing, 100191 China; 2 Research Center of Clinical Epidemiology, Peking University Third Hospital, No.49 North Garden Road, Haidian, Beijing, 100191 China

**Keywords:** Meniscectomy, Meniscus injury, Medial, Lateral, Risk factor

## Abstract

**Background:**

To evaluate the risk factor associated with total or subtotal meniscectomy for respective medial and lateral meniscus injury.

**Methods:**

The data of all the meniscus injured patients undergoing arthroscopy in our institute between January 15th, 2000 and December 31st, 2008 was collected and 6034 patients with 7241 injured menisci met the inclusion criteria. The mean patient age was 33.6 ± 14.9 years and there were 4785 males and 2456 females with 3568 medial and 3673 lateral menisci. The decision tree approach was applied to investigate the correlation of the tear type, the duration of complaint, age, gender, ACL rupture and total/subtotal meniscectomy for respective medial and lateral meniscus.

**Results:**

The tear type was associated with both medial (χ2 = 70.901, *P* < 0.001) and lateral (χ2 = 268.019, *P* < 0.001) total/subtotal meniscectomy. The strongest risk of total/subtotal meniscectomy of both medial and lateral meniscus tear was shown for the complex tear followed by the longitudinal, oblique, horizontal and radial tear of the medial meniscus and followed by horizontal, longitudinal, radial and oblique tear of the lateral meniscus. The risk of total/subtotal medial meniscectomy was significantly elevated for the patients with complex tear and the age of ≤40 years old (χ2 = 21.028, *P* < 0.001) and those with the oblique, horizontal or radial tear accompanied by ACL rupture (χ2 = 6.631, *P* = 0.01). Besides, the duration of complaint was also associated with total/subtotal meniscectomy of the medial longitudinal tear with ACL rupture (χ2 = 17.155, *P* < 0.001). On the other side, the risk of total/subtotal lateral meniscectomy was significantly elevated for the complex tear of the female patients (χ2 = 5.877, *P* = 0.015) with no ACL rupture (χ2 = 50.501, *P* < 0.001). The ACL rupture was associated with a decreased risk of total/subtotal meniscectomy for all the types of the lateral meniscus (complex: χ2 = 50.501, *P* < 0.001; horizontal: χ2 = 20.897, *P* < 0.001; oblique: χ2 = 27.413, *P* < 0.001; longitudinal and radial: χ2 = 110.85, *P* < 0.001).

**Conclusion:**

Analyzing data from a big sample available in an Asian patient database, we found different risk factors associated with total/subtotal meniscectomy for respective medial and lateral meniscus. Identifying patients at high risk for total/subtotal meniscectomy may allow for interventions after meniscus injury.

## Background

Meniscus injury is one of the most common sports injuries [1, 2]. As a vital part of the knee joint, the meniscus serves important roles including load bearing, shock absorption, stabilization, lubrication, nutrition of the articular cartilage and proprioception [3, 4]. Despite of meniscus repair and transplantation, total or subtotal meniscectomy was still inevitable for severe meniscus injury. Biomechanical studies have confirmed the decrease in intra-articular contact area and increase in the peak contact pressure after meniscectomy [5, 6], especially after total or subtotal meniscectomy, which significantly elevated the risk of osteoarthritis [7]. Thus identifying patients at high risk for total/subtotal meniscectomy may allow for interventions after meniscus injury.

Some risk factors have been confirmed to be associated with meniscus injuries [8, 9], including gender, sport, and type of exposure [5, 10, 11]. A large series of patients undergoing anterior cruciate ligament (ACL) reconstruction identified that increased age, male gender, and increased surgical delay were associated with a higher frequency and severity of meniscus injuries [12]. However, the correlation between the surgical option (partial or total meniscectomy or repair) and the patients’ parameters has been rarely studied.

In addition, the difference have been confirmed to exist in the injury characteristics between the medial and lateral meniscus [13, 14]. Dandy et al. [13] investigated 1000 symptomatic meniscus injury patients and found that there were significantly more medial meniscus tears in males than in females, there were more longitudinal tears than horizontal tears in the medial meniscus, and the most common tear type of the lateral meniscus is a longitudinal tear. Bergkvist [15] et al. reported 1375 cases of degenerative meniscal tears in 4096 arthroscopies, finding that there were more posterior horn tears in medial radial tears and more meniscal body tears in lateral radial tears. The difference of medial and lateral menisci in tear characteristics might affect the surgical option, especially the total/subtotal meniscectomy. So far, few study has evaluated the risk factor correlated with the total/subtotal meniscectomy, especially comparing the difference between medial and lateral meniscus.

The purpose of the present study was to evaluate the risk factor associated with total or subtotal meniscectomy for respective medial and lateral meniscus injury through the analysis of the 7241 meniscal tears. We hypothesized that the tear type, the duration of complaint, age, gender and ACL rupture might be associated with the risk of total/subtotal meniscectomy and the difference might exist between the medial and the lateral meniscus.

## Methods

### General information

The study was a retrospective study and was approved by the Medical Ethics Committee of Peking university third hospital (No: IRB00006761-2011097). A total of 6736 patients underwent arthroscopic meniscus treatment in our institute between January 15th, 2000 and December 31st, 2008. Those patients with discoid meniscus injuries, posterior cruciate ligament tears or collateral ligament tears were excluded thus 6034 patients with 7241 torn menisci were included in this study. The mean patient age was 33.6 ± 14.9 years and there were 4785 males and 2458 females with 3568 medial and 3673 lateral menisci.

There were 4037 patients diagnosed with ACL rupture and underwent ACL reconstruction at the time of meniscus surgery. The duration of complaint was recorded from the primary injury or the symptom onset of the patients with not clear trauma history. For detailed statistical analysis, the duration of complaint were divided into 0-28 days, 29-56 days, 57 day-1 year, 1-2 year and >2 years. In addition, the patients were also divided into 2 groups based on age ≤ 40 years and >40 years.

All patients were given spinal/epidural anesthesia with bupivacaine. All the surgeries were performed with arthroscopy (Smith-Nephew, Boston, MA, USA) according to a unified and standardized protocol. The joint was thoroughly examined by anteromedial and anterolateral portal using the 30 degree scope in sequential manner. The meniscal tear was confirmed by probing with the same principle of operation indication and technique referenced to the O’Connor’s classification [16]. The tear types of both medial and lateral meniscus was recorded as longitudinal, horizontal, oblique, radial, and complex. The surgeons removed all ruptured and offending meniscal tissue and left a stable and smooth rim of meniscus. When the degenerative changes and ruptures were present within the entire meniscus and the rupture reached as far as the synovial junction, total meniscectomy was performed. Excision of the pathological tissue was carried out with punch and mechanical shaver. In most of the cases partial meniscectomy and repair were kept as the first choice of treatment, preferable to subtotal or total meniscectomy. The meniscectomy was recorded as partial if any part of the meniscus was removed, leaving a minimum of two-thirds of the meniscal surface intact. More than one-third of the meniscal surface resection was recorded as subtotal [17, 18], most of which underwent posterior horn and mid-third resection with an intact rim and root attachments. All the data including the parameters of the patients and the management of all the meniscus tears were obtained from the medical records.

### Data analysis and statistics

All the data were analyzed using SPSS software (version 24.0; IBM Corporation, Armonk, New York). Two data sets were built from the source data set. Medial meniscus injured set involved patients with the diagnosis of medial meniscus tears while the lateral meniscus injured set involved patients with the diagnosis of lateral meniscus tears. For each set, the total or subtotal meniscectomy were treated as outcome events, while the partial meniscectomy or meniscus repair were considered as not event. Then the age was translated into binary variable of ≤40 years or >40 years; while the time to operation was translated into categorical variable. For the univariate analysis, all categorical variable such as age over 40, time slot to operation, gender, side, ACL injured were described with proportion and compared with Chi-square test.

Since the tear category was complicated, and interaction might exist between multiple variables. In order to make the results easier to interpret, the decision tree approach was applied to investigate the association between these factors and total/subtotal meniscectomy. The decision tree was set up by SPSS 24 and established by CHAID method. The minimum number of father and child nodes was 100 and 50, respectively, and the maximum tree depth is 3. A *P* value of less than 0.05 was considered statistically significant.

## Results

There were 1124 (31.5%) medial and 1413 (38.5%) lateral meniscus tears undergoing total/subtotal meniscectomies respectively with significant difference (χ2 = 15.47, *P* < 0.001). Besides, there were 1621 (45.5%) medial and 1937 (52.7%) lateral meniscus tears undergoing partial meniscectomies respectively with significant difference (χ2 = 37.754, *P* < 0.001). Significant more repairs of medial meniscus were performed than those of lateral meniscus (730 (20.5%) vs 323 (8.8%), χ2 = 198.194, *P* < 0.001).

The general information and the univariate analysis of the respective medial and lateral meniscus tears were shown in Table 1. The Chi-square test showed significant difference in the age, the duration of complaint and the tear type for both medial and lateral meniscus (*P* < 0.05). No significant difference was found in the injured side for both medial and lateral meniscus (n.s.).

**Table 1:** General Information and univariate analysis of Medial and Lateral Meniscus Tears

		Medial		Lateral	
Variable	Groups	Meniscus tears	Total/subtotal meniscectomy (%)	Meniscus tears	Total/subtotal meniscectomy (%)
Age	≤40	2525	825(32.7%)	2891	1056(36.5%)
	>40	1043	299(28.7%)	782	357(45.7%)
	χ2/*P*		χ2 = 5.489, *P* = 0.019*		χ2 = 21.652, *P* < 0.001*
Gender	Female	1243	391(31.5%)	1213	534(44%)
	Male	2325	733(31.5%)	2460	879(35.7%)
	χ2/*P*		χ2 = 0.002, *P* = 0.965		χ2 = 23.595, *P* < 0.001*
Side	Left	1734	523(30.2%)	1712	654(38.2%)
	Right	1834	601(32.8%)	1961	759(38.7%)
	χ2/*P*		χ2 = 2.81, *P* = 0.094		χ2 = 0.098, *P = 0.754*
ACL	Intact	1480	441(29.8%)	1724	895(51.9%)
	Rupture	2088	683(32.7%)	1949	518(26.6%)
	χ2/*P*		χ2 = 3.407, *P* = 0.065		χ2 = 248.09, P < 0.001*
Duration of complaint	0 – 28 days	310	93(30%)	404	131(32.4%)
	29-56 weeks	247	58(23.5%)	285	90(31.6%)
	57 d – 1 year	1680	513(30.57%)	1773	675(38.1%)
	1 – 2 year	462	149(32.3%)	439	170(38.7%)
	>2 years	869	311(31.5%)	772	347(44.9%)
	χ2/*P*		χ2 = 15.933, *P* = 0.003*		χ2 = 25.771, P < 0.001*
Tear types	Longitudinal	1624	504(31%)	826	272(32.9%)
	Radial	219	55(25.1%)	585	168(28.7%)
	Oblique	253	57(22.5%)	480	80(16.7%)
	Horizontal	408	83(20.3%)	507	197(38.9%)
	Complex	1064	425(39.9%)	1275	696(54.6%)
	χ2/*P*		χ2 = 72.426, P < 0.001*		χ2 = 270.585, P < 0.001*

*represents the significant difference (*P* < 0.05)

The percentage of total/subtotal meniscectomy for the patients ≤40 was more than those >40 for the medial meniscus tears while the opposite results were found for the lateral meniscus. Compared to the male patients with lateral meniscus tear, the total/subtotal meniscectomy was prone to the female patients with lateral meniscus tear (χ2 = 23.595, *P* < 0.001). No significant difference was found between medial meniscus injured patients accompanied by or without ACL rupture (n.s.) while more total or subtotal meniscectomy was found in the lateral meniscus injured patients with intact ACL than those with ACL rupture (χ2 = 248.09, *P < 0.001*).

**Fig. 1:**
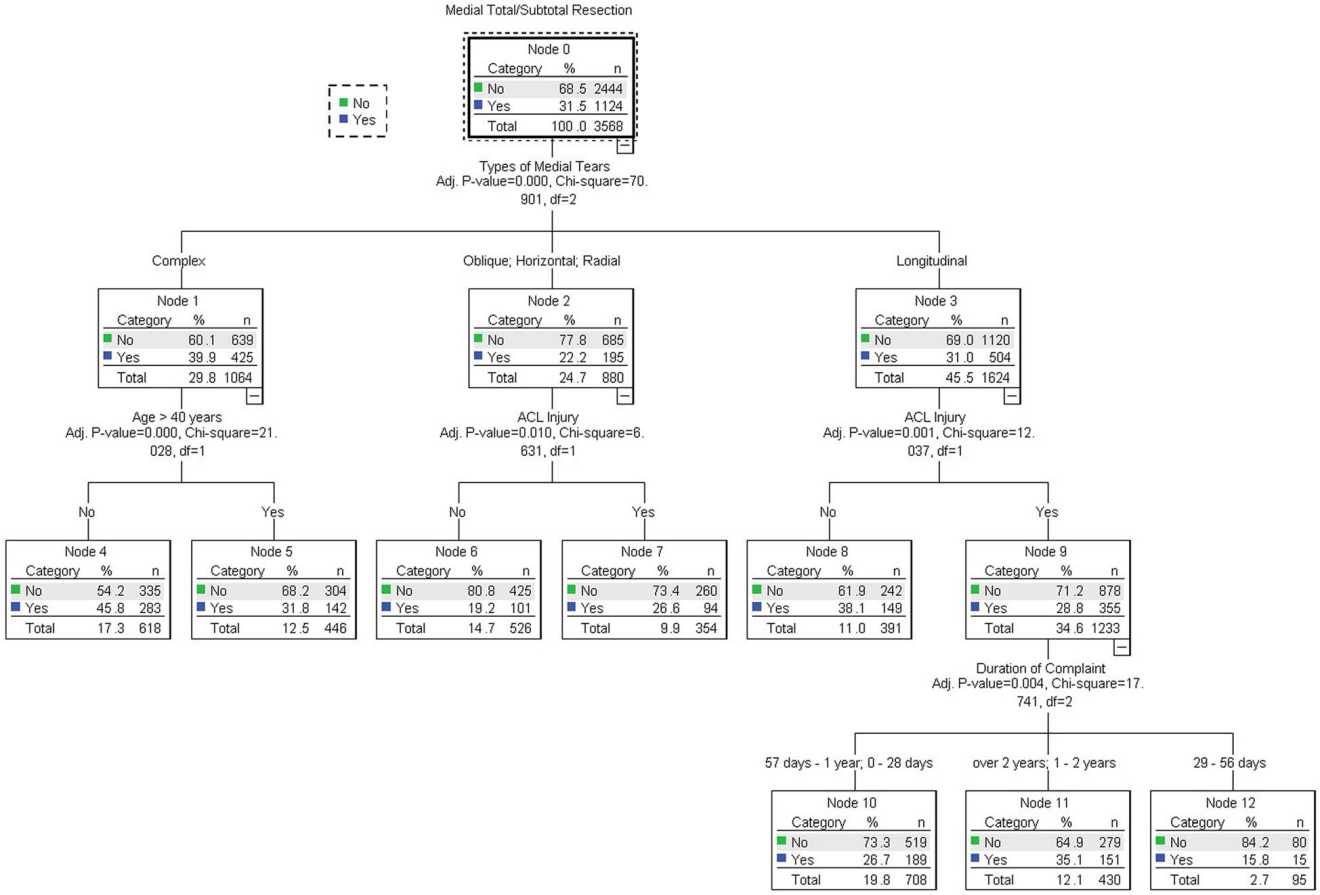
Results of the decision tree for the medial meniscus tear. The tear type was associated with medial total/subtotal meniscectomy (χ2 = 70.901, *P* < 0.001). The strongest risk of total/subtotal meniscectomy was shown for the complex tear (39.9%) followed by the longitudinal (31%) and oblique, horizontal and radial tear (22.2%). The risk of total/subtotal medial meniscectomy was significantly elevated for the complex tear with less than 40 years old (χ2 = 21.028, *P* < 0.001) and the oblique, horizontal or radial tear accompanied by ACL rupture (χ2 = 6.631, *P* = 0.01). Besides, the duration of complaint was associated with total/subtotal meniscectomy of the medial longitudinal tear accompanied by ACL rupture (χ2 = 17.741, *P* = 0.004)

The results of the decision tree for the medial meniscus tear was shown in Fig. 1 and the gains of nodes were shown in Table 2. The tear type was associated with medial total/subtotal meniscectomy (χ2 = 70.901, *P* < 0.001). The strongest risk of total/subtotal meniscectomy was shown for the complex tear (39.9%) followed by the longitudinal (31%) and oblique, horizontal and radial tear (22.2%). The risk of total/subtotal medial meniscectomy was significantly elevated for the complex tear with less than 40 years old (χ2 = 21.028, *P* < 0.001) and the oblique, horizontal or radial tear accompanied by ACL rupture (χ2 = 6.631, *P* = 0.01). Besides, the duration of complaint was associated with total/subtotal meniscectomy of the medial longitudinal tear accompanied by ACL rupture (χ2 = 17.741, *P* = 0.004).

**Table 2:** Gains for Nodes of medial meniscus tear

Node	Node		Gain		Response	Index
	N	Percent	N	Percent		
4	618	17.30%	283	25.20%	45.80%	145.40%
8	391	11.00%	149	13.30%	38.10%	121.00%
11	430	12.10%	151	13.40%	35.10%	111.50%
5	446	12.50%	142	12.60%	31.80%	101.10%
10	708	19.80%	189	16.80%	26.70%	84.70%
7	354	9.90%	94	8.40%	26.60%	84.30%
6	526	14.70%	101	9.00%	19.20%	61.00%
12	95	2.70%	15	1.30%	15.80%	50.10%

**Fig. 2:**
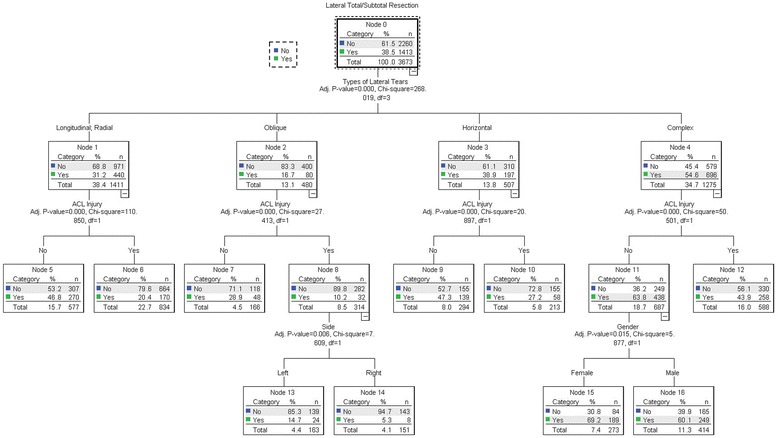
Results of the decision tree for the lateral meniscus tear. The tear type was also associated with the lateral total/subtotal meniscectomy (χ2 = 268.019, *P* < 0.001). The strongest risk of total/subtotal meniscectomy was shown for the complex tear (54.6%) followed by the horizontal (38.9%) and the longitudinal, radial (31.2%) and oblique tear (16.7%). The risk of total/subtotal lateral meniscectomy was significantly elevated for the complex tear of the female patients (χ2 = 5.877, *P* = 0.015) with no ACL rupture (χ2 = 50.501, *P* < 0.001). The risk of total/subtotal lateral meniscectomy was significantly elevated for patients with the oblique tear accompanied by ACL rupture in the left side (χ2 = 7.609, *P* = 0.006).The ACL rupture was associated with a decreased risk of total/subtotal meniscectomy for all the types of the lateral meniscus (complex: χ2 = 50.501, *P* < 0.001; horizontal: χ2 = 20.897, *P* < 0.001; oblique: χ2 = 27.413, *P* < 0.001; longitudinal and radial: χ2 = 110.85, *P* < 0.001)

The results of the decision tree for the lateral meniscus tear was shown in Fig. 2 and the gains of nodes were shown in Table 3. The tear type was also associated with the lateral total/subtotal meniscectomy (χ2 = 268.019, *P* < 0.001). The strongest risk of total/subtotal meniscectomy was shown for the complex tear (54.6%) followed by the horizontal (38.9%) and the longitudinal, radial (31.2%) and oblique tear (16.7%). The risk of total/subtotal lateral meniscectomy was significantly elevated for the complex tear of the female patients (χ2 = 5.877, *P* = 0.015) with no ACL rupture (χ2 = 50.501, *P* < 0.001). The risk of total/subtotal lateral meniscectomy was significantly elevated for patients with the oblique tear accompanied by ACL rupture in the left side (χ2 = 7.609, *P* = 0.006).The ACL rupture was associated with a decreased risk of total/subtotal meniscectomy for all the types of the lateral meniscus (complex: χ2 = 50.501, *P* < 0.001; horizontal: χ2 = 20.897, *P* < 0.001; oblique: χ2 = 27.413, *P* < 0.001; longitudinal and radial: χ2 = 110.85, *P* < 0.001).

**Table 3:** Gains for Nodes of lateral meniscus tear

Node	Node		Gain		Response	Index
	N	Percent	N	Percent		
15	273	7.40%	189	13.40%	69.20%	180.00%
16	414	11.30%	249	17.60%	60.10%	156.30%
9	294	8.00%	139	9.80%	47.30%	122.90%
5	577	15.70%	270	19.10%	46.80%	121.60%
12	588	16.00%	258	18.30%	43.90%	114.10%
7	166	4.50%	48	3.40%	28.90%	75.20%
10	213	5.80%	58	4.10%	27.20%	70.80%
6	834	22.70%	170	12.00%	20.40%	53.00%
13	163	4.40%	24	1.70%	14.70%	38.30%
14	151	4.10%	8	0.60%	5.30%	13.80%

## Discussion

Current meniscus repair techniques are only effective in treating lesions located in the peripheral vascularized region of the meniscus. Thus total or subtotal meniscectomy was still inevitable for the severe meniscus tears. Identifying patients at high risk for total/subtotal meniscectomy may allow for interventions after meniscus injury. However, few study has evaluated the risk factor associated with the total/subtotal meniscectomy, especially comparing the difference between medial and lateral meniscus. The purpose of the present study was to evaluate the risk factor of total or subtotal meniscectomy for respective medial and lateral meniscus injury. Analyzing data from a big sample available in an Asian patient database, we found different risk factors associated with total/subtotal meniscectomy for respective medial and lateral side. According to the results, meniscus tears should be treated as soon as possible to reduce the risk of total or subtotal meniscectomy, especially for the medial complex tear with age of ≤40, other medial tear type companied by ACL rupture and the lateral complex tear of female patients with intact ACL.

The tear type was significantly associated with both medial and lateral total/subtotal meniscectomy. The strongest risk of total/subtotal meniscectomy of both medial and lateral meniscus tear was shown for the complex tear followed by the longitudinal, oblique, horizontal and radial tear of the medial meniscus and followed by horizontal, longitudinal, radial and oblique tear of the lateral meniscus. As a special tear type, the complex tear usually combined with variety of tear types including oblique, horizontal and longitudinal tears, which significantly increased its risk of total/subtotal meniscectomy. The difference in the strength of the risk for the other tear types might be due to the difference of the anatomy and the dynamics between the medial and the lateral meniscus. Compared to the other tear types, the stability of the medial meniscus would be obviously decreased with the longitudinal tear, which was associated high risk of total/subtotal meniscectomy. On the other hand, there were 3 popliteomeniscal fascicles, which combined with the popliteus tendon form a peripheral hooplike attachment to the lateral meniscus but not to the medial meniscus. The popliteus complex provided both a static and dynamic connection between the lateral meniscus and the popliteus tendon and controlled the motion of the lateral meniscus during flexion and extension of the knee [19–22]. It was difficult to reconstruct both the static and dynamic connection for the lateral horizontal tear by meniscus repair. Thus the risk of total/subtotal meniscectomy was significant elevated.

Our study demonstrated another interesting result that increasing age was associated with a decreased risk of medial total/subtotal meniscectomy while the risk was elevated for the patients of ≤40 with complex tear. Previous studies have showed that increased age predicted intra-articular injuries [23–25]. However, few of them discussed about the correlation between increased age and the risk of meniscectomy. Kluczynski et al. [23] found that increased age predicted more meniscectomies in males. Ralles [26] et al. concluded that age was an independent predictor for medial meniscus injury. The discrepant findings from these researches might be due to the different data which include only the patients underwent ACL reconstructions. The present study included the patients both with and without ACL rupture. The protective effect of age on medial meniscus tears is probably due to the decrease in physical activity level after meniscus tears. In addition, a considerable proportion of the meniscus tears in elder patients were due to degeneration and the horizontal tear was usually only located at the posterior horn of medial meniscus. Thus partial meniscectomy was kept as the first choice other than total/subtotal meniscectomy for those patients, which might decrease the risk of total/subtotal meniscectomy.

As the most common associated injury, ACL rupture lead to abnormal joint forces and are associated with increased risk of injury to menisci, which normally transmit load and absorb shocks between the femur and tibia [27]. Our results concluded the idea of a significant decrease in the percentage of lateral total/subtotal meniscectomies with ACL rupture. This seems contradictory from the widely accepted view that the risk of meniscus tear and meniscectomy would be increased by the instability of ACL ruptured joint. A systematic review summarized data from 159 studies over the last 10 years which showed that meniscectomy was the most common method of treatment, performed overall in 65% of the meniscus tears [28]. Hagino et al. [29] reported lower percentage of meniscectomy, with 39% to 55% based on different surgical-timing group. However, Bellabarba et al. [30] summarized that the likelihood of a successful meniscal repair is enhanced significantly when combined with ACL reconstruction. Intra-articular bleeding caused by ACL reconstruction is also considered to provide a favorable environment for meniscal healing [31]. Thus total/subtotal meniscectomy would be decreased owing to the meniscus repair. Another reason of the risk reduction might be due to less sports activities with pain, giving way and loss of range of motion after ACL rupture, which partly prevented the existing meniscus tears from developing to a serious type. Compared to the isolated meniscus tears, those with concurrent ACL rupture experienced a completely different mechanical environment of the knee joint, which might affect the risk of total/subtotal meniscectomy. Further studies are needed in the future.

According to the results, the ACL rupture was confirmed to be a protective factor for the lateral meniscectomy but be a risk factor for the medial meniscectomy. The reason might be due to the difference of the biomechanics and the anatomy between medial and lateral meniscus. For one thing, the medial meniscus is less mobile than the lateral meniscus. Thus it is less suitable to follow the increased anterior and rotational translation of the tibia, especially into dynamic situations [24, 32]. Feucht [33] et al. demonstrated that with ACL injury, male patients, patients < 30 years and patients who sustained a contact injury had a high risk of major lateral meniscal tear. Arner [34] et al. reported greater translation with ACL deficiency in lateral meniscus compared with medial meniscus by using minimally invasive techniques that may explain the greater incidences of acute lateral meniscus tears and chronic medial meniscus tears. In the present study, most of the cases was chronic meniscus tears with the duration of complaint of more than 1 months. Thus the aggravation of the meniscus tear was prone to the medial meniscus. For another, the tears near the hiatus could produce a small and unstable meniscal bridge and the stripe could dislocate into the joint space and cause incarceration [35]. The incarcerated meniscus would severely destroy the normal force transmission structure of the knee and result in further damage. Thus the lateral meniscus tear worsen faster than the medial meniscus tear in the ACL intact knee.

In terms of the duration of complaint, the difference also existed between respective medial and lateral side. The duration of complaint was not associated with the lateral total/subtotal meniscectomy. However, the risk of total or subtotal meniscectomy increased significantly for the patients with medial longitudinal tear accompanied by ACL rupture. The reason might be due to the mechanical environment changes after ACL rupture, especially for the longitudinal tear as the most unstable meniscus injury. Previous studies have shown that lateral meniscal tears are commonly associated with acute ACL tears, while medial meniscal tears are often associated with chronic ACL tears [36, 37]. Bramilla et al. [32] selected 1069 consecutive patients who underwent primary ACL reconstruction, recommended that the reconstruction not be delayed more than a year after the injury, owing to the increased risk of medial meniscal tears. Ralles [26] et al. illustrated that patients who received surgery > 12 months after initial injury underwent stronger risk of injury in medial meniscus than those within 12 months. Chhadia et al. [24], who chose similar time ranges, showed an increase of medial meniscus tears at 6 months from the injury. They also found a strong association of prolonged time to surgery with decreased meniscus repair rate. Krutsch [38] et al. made a recommendation for an ACL reconstruction within 6 months after trauma to preserve the medial meniscus from resection, which is consistent with our results. However, all the above studies were performed for the meniscus patients with concurrent ACL rupture, which was also one of the import factors affecting the treatment option. In the present study, all the meniscus tear patients with or without ACL rupture were included. The results was parallel to the above studies but further distinguished the difference between the medial and the lateral meniscus.

To our knowledge, this is the first study evaluating the risk factors of total/subtotal meniscectomy for respective medial and lateral meniscus. In spite that the trend of prevalence of meniscus tears was well studied by many previous research, including gender, sport, and type of exposure [5, 10, 11], few study analyzed about surgical options and time-window about meniscus lesions, especially for respective medial and lateral side. The results of the present study offer insight into the correlation of those factors with total/subtotal meniscectomy for respective medial and lateral meniscus and was beneficial for the surgeon and the patient more focusing on the surgical strategy to preserve meniscus as much as possible.

There were still some limitations for the present study. First, the level of evidence was low due to the single center retrospective study. Despite being one of the largest sports medicine centers of the country, the differences in patients’ sources, diagnosis and treatment principle between institutes might affect the results. Besides, the meniscus tear type have not be classified by widely accepted classifications by Warren [39] or West [40] and the root tear (10 mm from the insertion site) have not be distinguished but was only recorded as the radial or oblique tear of the posterior horn before 2000. The meniscus root tear might be analyzed according to the data after 2010 with future studies. Furthermore, the inaccurate course of the disease might affect the results since the duration of complaint was used instead of the injury duration. Approximately one third patients suffered meniscus tear but without any trauma history and those meniscus tears might be due to some small unnoticed trauma or the degeneration.

## Conclusion

Analyzing data from a big sample available in an Asian patient database, we found different risk factors associated with total/subtotal meniscectomy for respective medial and lateral meniscus. Identifying patients at high risk for total/subtotal meniscectomy may allow for interventions after meniscus injury. Meniscus tears should be treated as soon as possible to reduce the risk of total or subtotal meniscectomy, especially for the medial complex tear with age of ≤40, other medial tear type accompanied by ACL rupture and the lateral complex tear of female patients with intact ACL.

## Abbreviation

ACL: Anterior cruciate ligament
